# Uniaxial Dynamic Compressive Behaviors of Hydraulic Asphalt Concrete under the Coupling Effect between Temperature and Strain Rate

**DOI:** 10.3390/ma13235348

**Published:** 2020-11-25

**Authors:** Rui Tang, Zhenpeng Yu, Guoqing Liu, Furong Li, Wenbin Tang

**Affiliations:** 1 Department of Civil Engineering and Engineering Mechanics, Columbia University, New York, NY 10027, USA; tr970518@163.com; 2 Department of Civil Engineering, School of Mechanics and Engineering Science, Shanghai University, Shanghai 200444, China; 3 Power of China Guiyang Engineering Corporation Limited, Guiyang 550081, China; liugq@hydrochinaguiyang.com.cn; 4 Key Laboratory of Failure Mechanism and Safety Control Techniques of Earth-Rock Dam of the Ministry of Water Resources, Nanjing 210024, China; lifurong@ycit.edu.cn; 5 College of Civil Engineering, Yancheng Institute of Technology, Yancheng 224051, China; twbin6688@126.com

**Keywords:** hydraulic asphalt concrete, strain rate, temperature effect, dynamic compression, experimental research

## Abstract

To investigate the compressive dynamic properties of hydraulic asphalt concrete under various temperatures, four temperatures and four strain rates have been set to perform the uniaxial compression experiments using hydraulic servo machine in this paper. The influence of temperature and strain rate on the failure modes, stress-strain curves and mechanical characteristic parameters of hydraulic asphalt concrete is analyzed and the results reveal that the failure modes and stress-strain curves have significant temperature effect. When the temperature is between −20 °C and 0 °C, the failure mode is dominated by brittle failure of asphalt binder, and hydraulic asphalt concrete shows obvious strain softening. With the addition of temperature, the failure modes of specimens are transferred from brittle failure to ductile failure since the asphalt changes from elastic-brittleness to viscoelasticity. Influenced by temperature effect, the compressive stress-strain curves of hydraulic asphalt concrete show strain hardening while the peak stress of hydraulic asphalt concrete is obviously decreased, and the variation coefficient of peak stress has a power relation with temperature. With successive increases in strain rate, the stress-strain curves of hydraulic asphalt concrete gradually are transferred from strain hardening to strain softening. The peak stress and stiffness modulus of specimens under compression gradually increase, and the dynamic increase factor of peak stress is linearly related with the logarithm value of strain rate after dimensionless treatment. In terms of the quantitative analysis of the experimental data, two relationship models of the coupling effect between temperature and strain rate are proposed. The proposed models have good applicability to the quantitative analysis of the experimental results in the manuscript. This paper offers important insights into the application and development of hydraulic asphalt concrete in hydraulic engineering.

## 1. Introduction

Hydraulic asphalt concrete (HAC) is a composite material made of mineral aggregate, bitumen, coarse aggregate and fine aggregate, which has the features of good impermeability, strong deformability and excellent working performance to be widely used as the impermeable core in the earth-rock embankment dams. The impervious cores prepared by hydraulic asphalt concrete become more competitive than traditional clay cores since hydraulic asphalt cores can be tailored by adjusting the bitumen content to local conditions and climate especially for locations where fine materials for clay cores are scarce [1]. There are nearly 200 asphalt concrete core embankment dams (ACED) that are under construction or have been built worldwide, with China, Austria, Germany and Norway being the countries where ACED have been most commonly used [2]. In engineering applications, hydraulic asphalt concrete is not only subjected to static loads like structural weight, but also suffers from dynamic loads such as earthquakes, wind, impact and explosions, especially the impermeable core in dams located in high altitude and high intensity area. At the same time, hydraulic asphalt concrete has a strong temperature sensitivity referring to something that the mechanical properties are significantly influenced by temperature. The stress-strain behaviors of hydraulic asphalt concrete used in dams are sensitive to the loading strain rate, temperature level, rate of decrease in temperature and confining pressure [1,3]. Though the bitumen contributes a lot to the performance of asphalt concrete, the asphalt mixture gradation also has an obvious effect. Because under various asphalt mixture gradations, the contact angle, the cohesive and adhesive strength of the contact surface between aggregates are different. Therefore, it is of great engineering and research significance to study dynamic behaviors of hydraulic asphalt concrete with a specific asphalt mixture gradation. The study exploring the coupling effect between the strain rate and temperature on the mechanical performance of hydraulic asphalt concrete provides important insights into engineering and research.

Previous studies have explored the temperature effect on asphalt concrete. Akhtarpour et al. [4], Feizi-khankandi et al. [5], Wang et al. [6] investigated the dynamic performance of hydraulic asphalt concrete employing shear tests, and the results reported that the shear modulus and shear strength of hydraulic asphalt concrete decrease with successive increase of temperature. S. Pirmohammad et al. [7] carried out three-point fracture tests and found that the fracture resistance of hydraulic asphalt concrete first increased and then below a certain temperature (−20 °C) decreased. Huang et al. [8] suggested that following the addition of temperature, the initial tangent modulus and failure strain of hydraulic asphalt concrete increases significantly employing experiments on the temperature sensitivity of asphalt concrete. Studies investigating the mechanical properties of hydraulic asphalt concrete were carried out using uniaxial compression tests and triaxial compression tests by Wang [9] and Zhang [10]. Li [11] investigated the low-temperature cracking performance of asphalt concrete using the combination of ductility test, bending beam rheometer (BBR) test, and asphalt composition analysis test. Chen et al. [12] performed shear performance tests on hydraulic asphalt concrete considering the temperature range of 0 °C–20 °C, and showed that the shear strength of hydraulic asphalt concrete decreased nonlinearly by an increase in temperature, especially the strain softening is obvious at 0 °C. Li [13] studied the mechanical performance of asphalt concrete at low temperatures through triaxial tests, and indicated that low temperatures could directly affect the working performance and deformation features of asphalt concrete. Pszczola [14] studied the strength properties of different asphalt mixtures and found that the type of bitumen is an important factor to low-temperature properties of asphalt concrete. Several investigations mentioned above have been only concerned with the mechanical properties of hydraulic asphalt concrete at room temperature or in a specific temperature range.

The strain rate effect of concrete refers to the meaning that the mechanical performance of concrete suffers obvious changes under the influence of loading strain rates. In terms of the research on the coupling effect between temperature and strain rate on ordinary asphalt concrete, Bekele [15] assessed the dynamic modulus of asphalt concrete at low temperatures utilizing an automatic non-contact resonance excitation method. Wang tested the triaxial compression dynamic performance of dense and porous asphalt concrete at various temperatures and strain rates and identified that the compressive strength and stiffness of asphalt concrete under dynamic loads exhibited distinct temperature effect. Ahmed [16] examined that the horizontal tensile strain and vertical strain of asphalt concrete layers showed significant temperature sensitivity by means of the experimental research on road asphalt concrete layers under dynamic loads. Ning [17] employed the hydraulic servo device to test the dynamic compressive features of hydraulic asphalt concrete at various temperatures and he reported that the failure modes of hydraulic asphalt concrete present distinct strain rate effect under various temperatures. Huang [8] analyzed the dynamic characteristics of asphalt concrete using a dynamic triaxial machine with an adjusted temperature control system. As the impermeable core in dams, hydraulic asphalt concrete has a relatively high bitumen content and excellent temperature sensitivity. The research to date has been concerned with the static and dynamic performance of ordinary asphalt concrete. While there is a general paucity of research investigating the dynamic performance of hydraulic asphalt concrete under compression. Therefore, a more comprehensive study on the coupling effect between temperature and strain rate on the dynamic performance of hydraulic asphalt concrete should be examined.

This article reports the experimental studies on the coupling effect between temperature and strain rate on the compressive dynamic performance of hydraulic asphalt concrete where four temperatures and four strain rates are set out, employing the hydraulic servo machine. The failure modes, stress-strain curves and mechanical features parameters of hydraulic asphalt concrete at various loading conditions are compared and analyzed. A combined qualitative and quantitative methodological approach is used to analyze the mechanical performance of hydraulic asphalt concrete under the coupling effect between temperature and strain rate. The research results make an important contribution to the field of engineering application and development of hydraulic asphalt concrete.

## 2. Experimental Program

### 2.1. Mix Ratio and Sample Preparation

**Figure 1:**
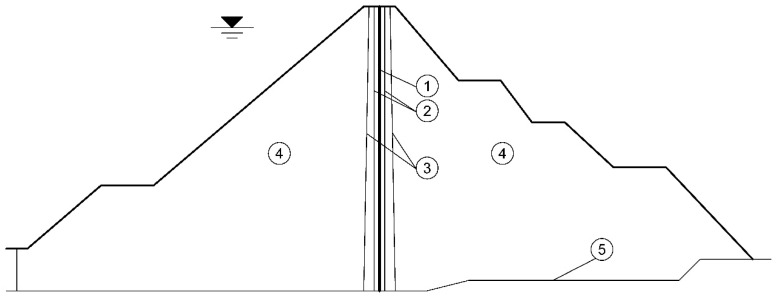
Cross-section of the asphalt concrete core embankment dam. (1) hydraulic asphalt concrete core; (2) fine transition zone; (3) coarse transition zone; (4) rockfill; (5) natural ground surface.

This article examines the uniaxial compressive dynamic performance of hydraulic asphalt concrete through experiments. Hydraulic asphalt concrete partially replaces the cement in ordinary concrete with mineral fillers and bitumen as binders. The asphalt mastic is considered as the combination of the aggregates with diameters smaller than 1.18 mm and the bitumen binder. While the ordinary asphalt concrete can be treated mixing the asphalt mastic and the aggregates with diameters larger than 1.18 mm [18]. By comparison with ordinary asphalt concrete used in pavements, hydraulic asphalt concrete has higher impermeability and elasticity because the optimal bitumen content of hydraulic asphalt concrete accounts for 6.5–8.5% of the total mix weight [19], higher than that of in ordinary asphalt concrete which is 5–5.3% [20,21]. Table 1 presents the mix ratio of ordinary asphalt concrete and hydraulic asphalt concrete calculated on the basis of the data in literatures [19,22] and the “Test code for hydraulic asphalt concrete” (DL/T 5362-2018) [23]. The bitumen binder type used is SBS modified bitumen while coarse aggregates are basalt and fine aggregates consist of limestone. The impervious core prepared by hydraulic asphalt concrete is located in the cross-section of the asphalt concrete embankment dams ACED, as shown in Figure 1. The prime function of the transition zone or filter zone in Figure 1 is to retain impervious core materials against movement into the rockfill made up of porous materials.

According to “Standard Test Methods of Bitumen and Bituminous Mixtures for Highway Engineering” (JTG E20-2011) [24] and related literature on the dynamic behaviors of asphalt concrete at various temperatures [1,9], the samples in the uniaxial compression tests are usually cylinders and prisms. The test results exhibited in the conference show that the effects of geometry do not affect the stress-strain behaviors of asphalt concrete under uniaxial compression [25]. And there is a common pattern of stress-strain curves identified into three stages: initial bottom concave part, an ascending branch followed by a descending branch [25]. Limited by the hydraulic servo device, according to “Test code for hydraulic asphalt concrete” [23] and the sample processing method that the cylinder cores are cut into cube specimens using the laboratory saw [26], the hydraulic asphalt concrete specimens are processed as cubes with side length of 100 mm. Firstly, the fine aggregate and coarse aggregate are placed in the oven for more than 4 h. Then the bitumen is added into and mixed the mixture thoroughly. The uniformly mixed specimens are placed in the oven for 2 h under compaction to keep impacted. The final step is to take specimens out of the oven and cure them at room temperature (25 °C) for 24 h before they are demoulded for testing. The volumetric properties of hydraulic asphalt concrete are presented in Table 2.

### 2.2. Loading Conditions

The test temperature range should be set to illustrate the temperature effect on the compressive dynamic behaviors of hydraulic asphalt concrete. Xue et al. [27] clearly reveal that the temperature of some mountains in winter where embankment dams are located could be lower than −20 °C and the results from Adam [28] show that the temperature range of asphalt concrete facing of embankment dams could reach −12 °C–45 °C by the means of the temperature monitoring of the asphalt concrete facing of a Dlouble Strane (Loucna nad Desnou, Czech Republic) pumped storage hydroelectric plant. According to the “Test code for hydraulic asphalt concrete” (DL/T 5362-2018) [23] as well as the consideration of the actual temperature range of asphalt core rockfill dams revealed in references mentioned above [27,28], four temperatures are set, including −20 °C, 0 °C, 25 °C and 45 °C. Depending on the method in reference [27], these specimens selected for low-temperature tests are placed in the industrial refrigerators for 48 h at −20 °C and 0 °C, respectively, to ensure that the specimens reach the set temperature and are fully uniform in temperature. At the same time, these specimens for high-temperature tests are placed in the oven for 48 h at 45 °C to ensure that the specimens reach the set temperature and are fully uniform in temperature while the specimens for room temperature tests (25 °C) are placed indoors for 48 h. All specimens used in the paper are then immediately taken out to complete required tests. Due to the relatively short duration of the tests, the temperature change of the specimens during the tests is relatively small which could be considered that the test results are uninfluenced by those minor changes. Therefore, the influence law of temperature on hydraulic asphalt concrete under compression could be analyzed. For the study of the dynamic behaviors of concrete, Shang [29] defines the range of loading strain rates for static load and seismic action as 1.0 × 10^−6^/s~1.0 × 10^−5^/s, 1.0 × 10^−3^/s~1.0 × 10^−2^/s, separately. Considering the magnitude of earthquake action, four strain rates, including 10^−5^/s, 10^−4^/s, 10^−3^/s and 10^−2^/s, are set out in the study to examine the compressive dynamic behaviors of hydraulic asphalt concrete, where 10^−5^/s is the static loading strain rate while 10^−4^/s, 10^−3^/s and 10^−2^/s are dynamic loading strain rates. In terms of the randomness and discreteness of hydraulic asphalt concrete and the requirements of the uniaxial compression tests in the “Test code for hydraulic asphalt concrete”, three specimens are set for each loading condition to pick the average of experimental values for the requirements of analysis. There are 16 loading conditions with a total of 48 specimens used in the paper.

### 2.3. Test Equipment and Loading Schemes

**Figure 2:**
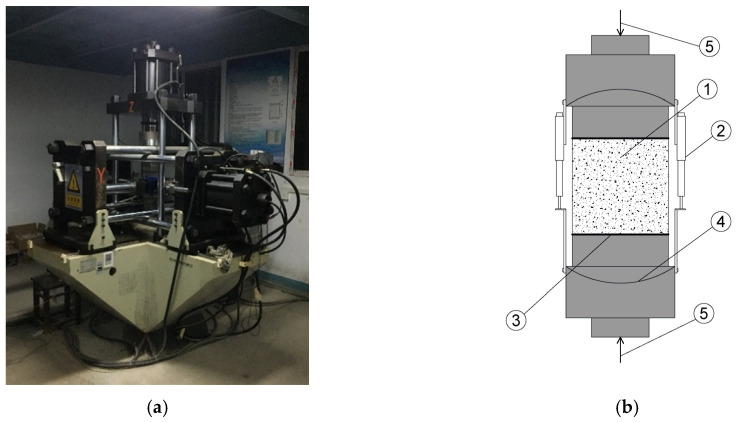
Diagrams of the test device and loading process. (**a**) loading test device; (**b**) schematic loading diagram of uniaxial compression tests: (1) concrete specimen; (2) load and displacement sensors (LVDT); (3) friction-reducing layers; (4) spherical hinges; (5) loading directions.

Hydraulic servo device is prepared to conduct the uniaxial compressive dynamic behaviors experiments of hydraulic asphalt concrete, as shown in Figure 2. The set of independent load and displacement sensors (LVDT) are equipped in the device. Especially, the maximum range of the load sensors is 100 tons and the maximum error is ±1% of the measured value while the maximum error of the displacement sensor is ±0.0005 mm. The measurement accuracy and error range of the sensors meet the test requirements. The stress value of hydraulic asphalt concrete is obtained through the ratio of the tested load of specimens originated from load sensors to the loading surface area. The strain value in the paper is measured by the displacement sensors and independent deformation acquisition system equipped in the hydraulic servo device during the loading process. The strain values generated in two sources are collected and calibrated synchronously by the comparison of each other.

**Figure 3:**
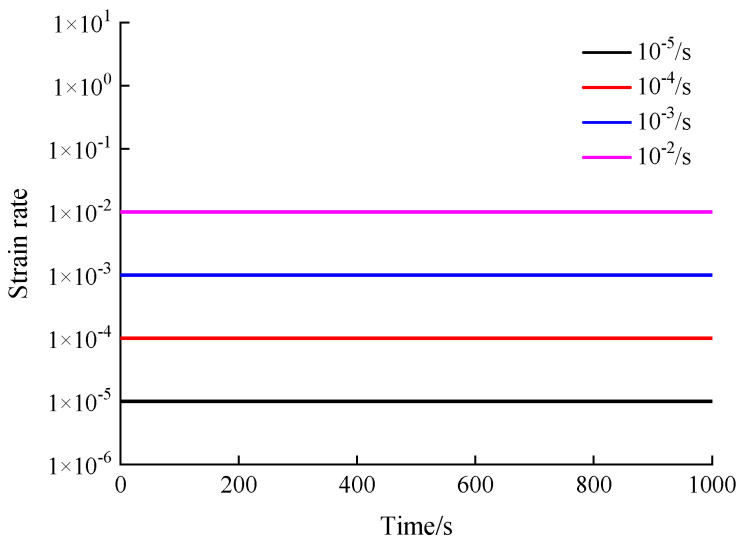
Schematic plot showing loading strain rates over time.

The uniaxial compression dynamic tests apply displacement-controlled method with the loading strain rates 10^−5^/s, 10^−4^/s, 10^−3^/s and 10^−2^/s in this manuscript. In detail, the displacement change value at every time is constant under the control of the device. Since all samples are prepared as cubes with the side length of 100 mm, and the strain is equal to the value of the displacement change value divided by the initial size of samples, the strain rate during the loading process could keep stable by the displacement-controlled method. The schematic plot showing loading strain rates over time is presented in Figure 3. The ordinate values of different lines are four loading strain rates controlled by the device. A combination of three layers of polyethylene plastic film sandwiched with mechanical butter is utilized to control the impact of the friction between the loading surface of the equipment and the interface of the specimen, which can satisfy the requirements of the tests.

## 3. Experimental Results and Analysis

### 3.1. Compression Failure Modes of Hydraulic Asphalt Concrete

**Figure 4:**
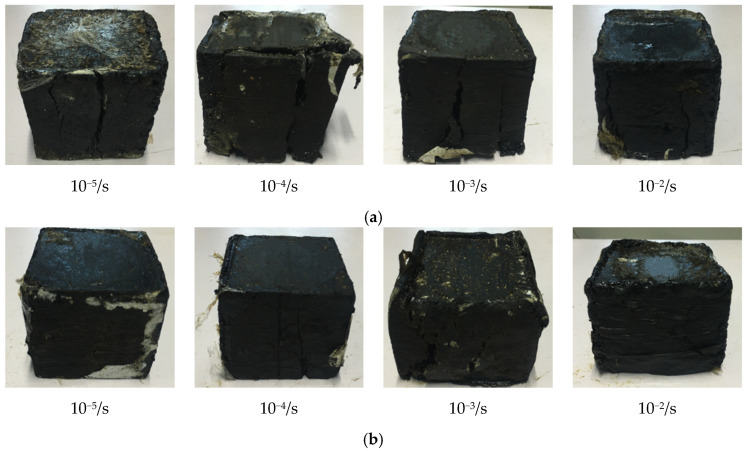

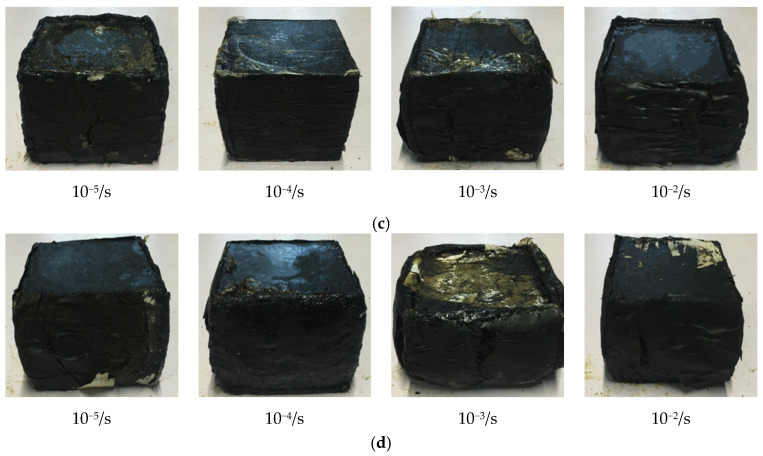
Failure modes of hydraulic asphalt concrete at various temperatures. (**a**) temperature −20 °C; (**b**) temperature 0 °C; (**c**) temperature 25 °C; (**d**) temperature 45 °C.

Depending on the test schemes of temperature effect on the compressive dynamic behaviors of hydraulic asphalt concrete, the hydraulic servo device is employed to obtain the compression failure modes of hydraulic asphalt concrete under various loading cases, as can be seen in Figure 4. In terms of the macro perspective, the coupling effect between temperature and strain rate on the compressive dynamic behaviors of hydraulic asphalt concrete is investigated.

It can be seen in Figure 4 that the integrity of hydraulic asphalt concrete after compression failure is relatively good without slags falling off the specimens, which indicates that the bitumen is strongly bonded with the aggregates. The uniaxial compressive failure mechanism of hydraulic asphalt concrete is explored. The compressive strain is formed in the axial direction of specimen under vertical loads. Meanwhile, the tensile strain develops perpendicular to the loading direction influenced by Poisson ratio effect. Following the addition of vertical loads, the tensile strain increases until reaching the ultimate value of hydraulic asphalt concrete, then the specimen is broken with cracks parallel to the loading direction.

Failure modes of hydraulic asphalt concrete at −20 °C from Figure 4a mainly show brittle fracture of bitumen binder, with small compression deformation along the loading direction as well as vertical cracks formed on free surfaces of specimens. With successive increase in strain rate, the number and size of cracks get higher. The vertical cracks are formed cross upper and lower edges at the strain rates 10^−5^/s and 10^−4^/s, and the width of cracks is large as well as the fracture point of cracks is smooth. The reason is explained that bitumen exhibits better elasticity and lower plastic deformability at low temperatures. The deformation of specimens is mainly the elastic deformation of aggregates and the elastoplastic deformation of the bitumen in the initial stage of compression loads. The micro cracks occur at the interfaces between bitumen and aggregates due to different characteristics of two materials. By an increase in compression load, the cracks develop vertically since the specimens are subjected to uniform stress and destroyed finally. When the strain rate is high, there is not enough time for micro cracks to develop and the stress state cannot be formed uniformly. In the end, the length and width of cracks are relatively small when the specimen is broken.

When it comes to the failure modes of hydraulic asphalt concrete at 0 °C temperature, the failure modes of specimens are mainly brittle failure of the bitumen binder at the strain rates 10^−5^/s and 10^−4^/s. And the overall deformation of specimens is relatively small and uniform vertical cracks are formed in the free surfaces. With successive increase in the strain rate, the failure modes of specimens change significantly. The compressive strain develops larger but the penetration fracture failure mode is not formed. Besides, the compressed transverse stripes appear on the free surfaces when the compressive effect of aggregates become more distinct. It can be explained that the compressive capacity of bitumen binder is far less than that of aggregates because hydraulic asphalt concrete is taken as the composite material. Under the low loading strain rate, the stress can be transferred to aggregates through bitumen binder and then aggregates suffer loads to produce elastic deformation, forming the relatively small compressive strain. However, following the addition of strain rate, the stress does not transfer along the uniform distribution path sufficiently. As a cementitious material, the bitumen binder suffers most loads directly. At temperature 0 °C, the performance of bitumen changes from elasticity and brittleness at temperature −25 °C to viscoelasticity while the deformability of hydraulic asphalt concrete is significantly improved. At the same time, the cracks on the free surfaces of the specimen are less and relatively inconspicuous when the specimen is broken, as displayed in Figure 4b.

When the temperature rises to 25 °C and 45 °C, the failure modes of hydraulic asphalt concrete all exhibit notable plastic deformation with the typical ductile failure of bitumen binder. The failure modes are presented in Figure 4c,d. The failure modes at the strain rate 10^−5^/s display that the vertical cracks are formed obviously on the free surfaces of specimens, accompanied by compressed transverse stripes. The number of cracks after failure decreases as well as the reduced length and width of cracks by an increase in the strain rate. And the horizontal stripes become more distinct. The transferred performance of bitumen at various temperatures is taken to suggest the reason. Bitumen shows notable viscoelasticity and large deformability at high temperature. Therefore, the bitumen binder produces greater plastic deformation, influence by strain rate and high temperature.

### 3.2. Uniaxial Compression Stress-Strain Curves

According to the experimental data generated from the test scheme mentioned above, the uniaxial compression stress-strain curves of hydraulic asphalt concrete under various loading conditions are generated. The effects of strain rate and temperature on the compressive stress-strain curves are examined by comparison and analysis.

#### 3.2.1. Temperature Effect of Stress-Strain Curves

**Figure 5:**
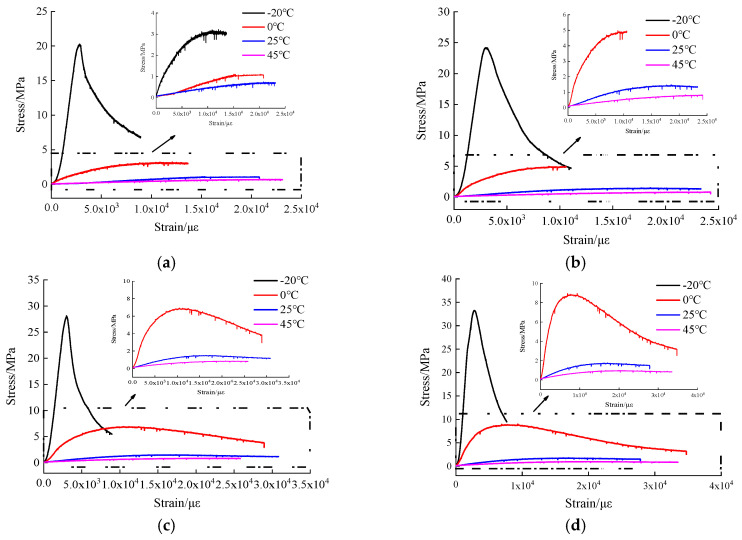
Stress-strain curves of hydraulic asphalt concrete under different strain rates. (**a**) strain rate 10^−5^/s; (**b**) strain rate 10^−4^/s; (**c**) strain rate 10^−3^/s; (**d**) strain rate 10^−2^/s.

The influence of temperature effect on stress-strain curves of hydraulic asphalt concrete under various strain rates is shown in Figure 5.

To facilitate visual analysis, the compressive stress-strain curves above 0 °C are partially enlarged. When looking at Figure 5, the development trend of stress-strain curves of hydraulic asphalt concrete at various temperatures is similar, showing good continuity and smoothness. The compressive stress-strain curves under different loading conditions could be divided into three stages, including the initial compression stage, increasing stage and descending stage.

At the initial compression stage, the compressive strain increases continuously while the stress increases slowly because the internal pores of the specimen are firstly compressed due to the existence of apparent defects of specimens and relaxation effect of bitumen. For the increasing stage, when the temperature is −20 °C, the specimen produces relatively small deformation since bitumen is an elastic-brittle material. The stress increases linearly with the strain, and the curve has an obvious peak point, meanwhile the strain is small before the stress reaches the peak value. When the temperature rises to 0 °C, the mechanical performance of bitumen changes from elasticity and brittleness to viscoelasticity influenced by temperature, which makes elastoplastic deformation in the specimen. The stress is increased nonlinearly with the strain. Bitumen exhibits strong viscoelasticity at temperatures 25 °C and 45 °C, and the stress reaches the peak value in a nonlinear growth way. After the stress reaches the peak value, the stress-strain curve enters the descending stage. When temperatures are −20 °C and 0 °C, the stress decreases rapidly by an increase in the strain, displaying distinct strain softening. The specimens present the features of brittle failure. Meanwhile, the stress decline of specimens at temperature −20 °C is higher than that at temperature 0 °C. It indicates that the lower the temperature, the more distinct the strain softening of hydraulic asphalt concrete. Considering the specimens at temperatures 25 °C and 45 °C, the stress tends to be stable with the addition of the strain after the peak point. Hydraulic asphalt concrete shows strain hardening, accompanied by features of ductile failure. The reason for this observation can be illustrated that bitumen binder behaves elastic-brittle under low temperature conditions. The specimens occur brittle failure with small deformation. As the temperature rises, the mechanical properties of bitumen binder change from elastic brittleness to viscoelasticity. The softened bitumen enhances the fluidity of hydraulic asphalt concrete, resulting in rapid increase in the deformation of specimens under compression. Therefore, the specimens show notable ductile failure.

#### 3.2.2. Strain Rate Effect of Stress-Strain Curves

**Figure 6:**
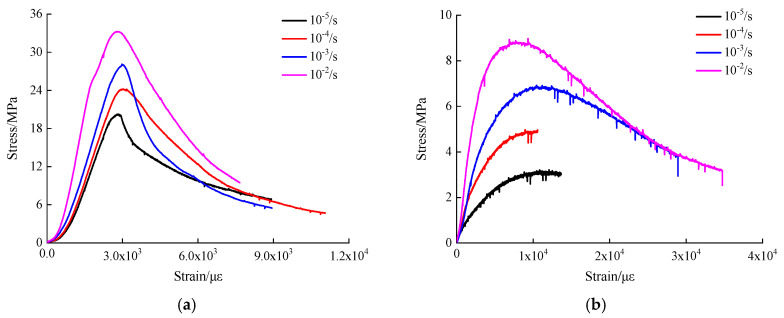

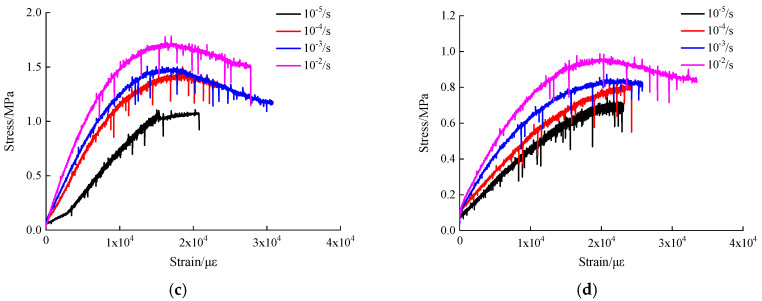
Stress-strain curves of hydraulic asphalt concrete under different temperatures. (**a**) temperature −20 °C; (**b**) temperature 0 °C; (**c**) temperature 25 °C; (**d**) temperature 45 °C.

The influence of strain rate effect on stress-strain curves of hydraulic asphalt concrete at the same temperature is investigated through stress-strain curves shown in Figure 6.

It can be seen in Figure 6 that the stress-strain curves of hydraulic asphalt concrete are similar at one certain temperature. The stress-strain curves include five parts, the initial bottom part, linear part, nonlinear part, peak and stretches part, and descending part in the reference [30]. On the basis of the stage division of curves in ordinary asphalt concrete, the stress-strain curves of hydraulic asphalt concrete are simplified into three stages in this paper, including the initial compression stage, the increasing stage and descending stage. The stress first increases to reach the peak point and then decreases with the increase of the strain at static strain rate. As the loading strain rate increases, the slope of the increasing stage and peak stress of stress-strain curves rise significantly. When the strain rate is 10^−2^/s, the specimens all show notable strain softening under four temperatures. The specimens produce large plastic deformation and ductile failure occurs. When the strain rates are 10^−5^/s and 10^−4^/s, the stress tends to be stable with the increase of the strain after the peak stress, presenting distinct strain hardening. According to the results mentioned above, the greater the strain rate, the more obvious the strain softening of specimens. The mechanism can be explored that when the strain rate is low, the stress of hydraulic asphalt concrete develops uniformly and is transferred to aggregates, so that the aggregates are subjected to large pressure. The aggregates have sufficient time for stress redistribution to form a more stable structure. Therefore, the stress decreases slowly by an increase in the strain after the stress reaches the peak value. The elasticity of bitumen has important influence on the elastic properties and stiffness modulus of specimens. The increase in temperature leads to the change of bitumen properties, which softens the bitumen binder and improves the flow of hydraulic asphalt concrete [31]. At the same time, the stiffness modulus of hydraulic asphalt concrete increases by an increase in the strain rate. The main reason is that the dynamic strain rate has certain stiffening effect on the bitumen binder, aggregates. The hardening effect of hydraulic asphalt concrete is more distinct with an increasing strain rate [9].

### 3.3. Peak Compressive Stress

**Figure 7:**
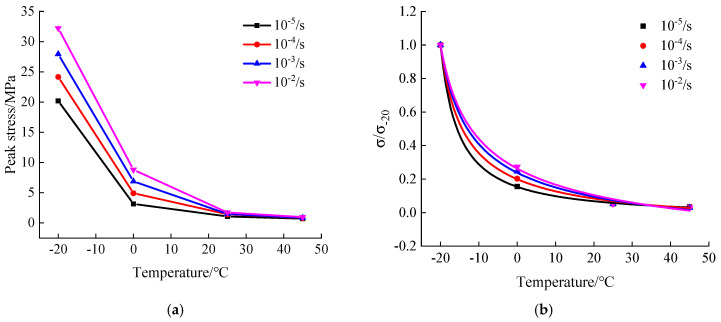
Relation between temperature and peak stress of hydraulic asphalt concrete. (**a**) peak stress; (**b**) variation coefficient.

**Figure 8:**
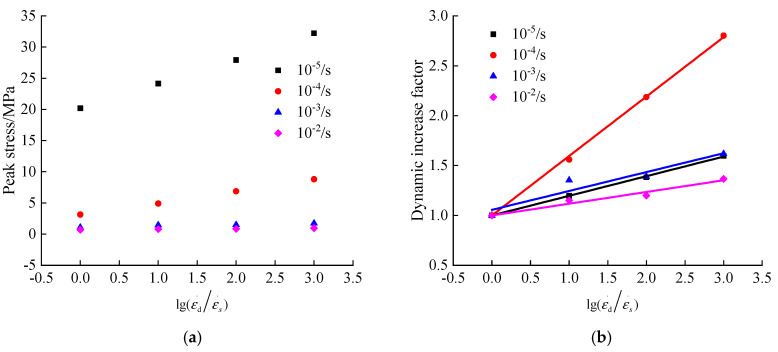
Relation between strain rate and peak compressive stress of hydraulic asphalt concrete. (**a**) peak stress; (**b**) dynamic increase factor.

The peak stresses extracted from uniaxial compression stress-strain curves of hydraulic asphalt concrete under various loading conditions are presented in Figure 7 and Figure 8. Since the experimental data collected during the test process is not smooth enough, the peak stresses extracted directly is not accurate. The data of stress-strain curves is smoothed to obtain the peak compressive stress of hydraulic asphalt concrete employing smoothdata function in MATLAB.

As can be seen in Figure 7a, with increasing temperature, the peak compressive stress of hydraulic asphalt concrete gradually decreases under the same strain rate and the decrease of peak stress is reduced. When the temperature rises to 0 °C, the peak compressive stress of hydraulic asphalt concrete at various strain rates is less than 10 MPa. When the strain rate is 10^−5^/s, the temperature is increased from −20 °C to 45 °C, and the peak compressive stress decreases from 20.185 MPa to 0.700 MPa by 96.5%. When the strain rate is 10^−4^/s, the temperature is increased from −20 °C to 45 °C, and the peak compressive stress decreases from 24.146 MPa to 0.805 MPa by 96.6%. When the strain rate is 10^−3^/s, the temperature is increased from −20 °C to 45 °C, and the peak compressive stress decreases from 27.920 MPa to 0.839 MPa by 96.9%. When the strain rate is 10^−2^/s, the temperature is increased from −20 °C to 45 °C, and the peak compressive stress decreases from 32.216 MPa to 0.956 MPa by 97.0%. The studies mentioned above show that the decline percentage of the peak compressive stress of hydraulic asphalt concrete influenced by temperature effect is basically the same with an increasing strain rate, which reveals that the peak stress is little influenced by the strain rate effect.

Taking account of the temperature effect on the peak compressive stresses of hydraulic asphalt concrete under various loading conditions, the peak compressive stress at temperature −20 °C is taken as the reference value $\sigma_{-20}$, while $\sigma_{0},\;\sigma_{25}$ and $\sigma_{45}$ are taken as the analyzed values. The mathematical regression analysis of experimental data is carried out by MATLAB to generate the mathematical expressions of the influence of temperature effect on the peak stress ratio of hydraulic asphalt concrete, as presented in Figure 7b and Equations (1)–(4):

Strain rate 10^−5^/s:

(1) $$\begin{array}{c} \sigma /\sigma_{-20}=5.749\times {\left(T+25\right)}^{-1.068}-0.03045\;R^{2}=0.9998 \end{array}$$

Strain rate 10^−4^/s:

(2) $$\begin{array}{c} \sigma /\sigma_{-20}=3.974\times {\left(T+25\right)}^{-0.7922}-0.1104\;R^{2}=0.9993 \end{array}$$

Strain rate 10^−3^/s:

(3) $$\begin{array}{c} \sigma /\sigma_{-20}=3.192\times {\left(T+25\right)}^{-0.5785}-0.2576\;R^{2}=0.9988 \end{array}$$

Strain rate 10^−2^/s:

(4) $$\begin{array}{c} \sigma /\sigma_{-20}=2.961\times {\left(T+25\right)}^{-0.4678}-0.3941\;R^{2}=0.9981 \end{array}$$

It can be analyzed in Figure 7b and Equations (1)–(4) that the peak stress and temperature of hydraulic asphalt concrete under four strain rates exhibit a power function relation, and the proposed equations are applicable which better describe the variation coefficient of the peak stress under various temperatures. As can be seen in the regression curves, following the addition of the temperature the peak compressive stress decreases, approaching zero.

Damage patterns and mechanical properties of concrete present various forms under dynamic loading, which is called the strain rate effect and is characterized by the dynamic increase factor [32]. The dynamic increase factor $\alpha_{DIF}\;$is introduced to analyze the strain rate effect on the peak compressive stress of hydraulic asphalt concrete, as displayed in Equation (5) and the relation between the strain rate and peak stress of hydraulic asphalt concrete is presented in Figure 8a:

(5) $$\begin{array}{c} \alpha_{DIF}=\frac{\sigma_{d}}{\sigma_{s}} \end{array}$$

where $\alpha_{DIF}$ denotes the dynamic increase factor of the peak compressive stress of concrete, $\sigma_{d}\;\mathrm{and}\;\sigma_{s}\;$are the peak compressive stress at dynamic and static loading strain rate, respectively.

It can be derived in Figure 8a that the peak compressive stress of hydraulic asphalt concrete increases significantly by an increase in the strain rate. When the temperature is −20 °C, the peak compressive stress of hydraulic asphalt concrete increases from 20.19 MPa at static strain rate to 32.22 MPa at dynamic strain rate 10^−2^/s, with an increase of 59.60%. When the temperature is 0 °C, 25 °C and 45 °C, the peak compressive stresses of hydraulic asphalt concrete increase from 3.14 MPa, 1.06 MPa and 0.70 MPa at static strain rate to 8.81 MPa, 1.72 MPa and 0.96 MPa at dynamic strain rate 10^−2^/s, which are increased by 2.80 times, 1.62 times and 1.37 times, respectively. Overall, these results indicate that the peak compressive stress of hydraulic asphalt concrete is most affected by the strain rate at temperature 0 °C.

To quantitatively analyze the dynamic compressive performance of hydraulic asphalt concrete, the dynamic increase factor of the peak compressive stress is considered to be linearly related with the logarithmic value of the strain rate ratio after non-dimensionless treatment in related literature [33], as shown in Equation (6). The relation between the dynamic increase factor and strain rate is obtained in Equations (7)–(10) and Figure 8b, by means of the mathematical regression analysis performed by Equation (6).

(6) $$\begin{array}{c} \alpha_{DIF}=a+b\log\left(\frac{\dot{\varepsilon_{d}}}{\dot{\varepsilon_{s}}}\right) \end{array}$$

At −20 °C temperature:

(7) $$\begin{array}{c} \alpha_{DIF}=1+0.1965\times \log\left(\frac{\dot{\varepsilon_{d}}}{\dot{\varepsilon_{s}}}\right)\;R^{2}=0.9993 \end{array}$$

At 0 °C temperature:

(8) $$\begin{array}{c} \alpha_{DIF}=1+0.5955\times \log\left(\frac{\dot{\varepsilon_{d}}}{\dot{\varepsilon_{s}}}\right)\;R^{2}=0.9992 \end{array}$$

At 25 °C temperature:

(9) $$\begin{array}{c} \alpha_{DIF}=1.056+0.1891\times \log\left(\frac{\dot{\varepsilon_{d}}}{\dot{\varepsilon_{s}}}\right)\;R^{2}=0.9150 \end{array}$$

At 45 °C temperature:

(10) $$\begin{array}{c} \alpha_{DIF}=1+0.1174\times \log\left(\frac{\dot{\varepsilon_{d}}}{\dot{\varepsilon_{s}}}\right)\;R^{2}=0.9625 \end{array}$$

As Figure 8b and Equations (7)–(10) show, the proposed equations are applicable to quantitatively describe the linear relation between the dynamic increase factor of uniaxial peak compressive stress of hydraulic asphalt concrete and the logarithmic value of the strain rate ratio after non-dimensionless treatment under four temperatures.

### 3.4. Deformation Parameters of Hydraulic Asphalt Concrete

For the analysis of the deformation parameters of hydraulic asphalt concrete, the influence of strain rate effect and temperature effect on the deformability could be examined from two perspectives of stiffness modulus and peak strain.

#### 3.4.1. Stiffness Modulus

Since the stiffness modulus is one of the most important parameters in the stress-strain curves, it can reflect the deformability of concrete. The effect of temperature and strain rate on the deformability can be investigated by analyzing the stiffness modulus extracted from stress-strain curves.

In order to accurately calculate the stiffness modulus of hydraulic asphalt concrete, based on the calculation method used in reference [33], the ratio of 50% of the peak stress value to the corresponding strain value is employed as the stiffness modulus and displayed in Equation (11):

(11) $$\begin{array}{c} S=\frac{\sigma_{0.5}}{\varepsilon_{0.5}} \end{array}$$

where S is the stiffness modulus of hydraulic asphalt concrete, $\sigma_{0.5}$ refers to the 50% peak stress value and $\varepsilon_{0.5}$ represents the corresponding strain value.

**Figure 9:**
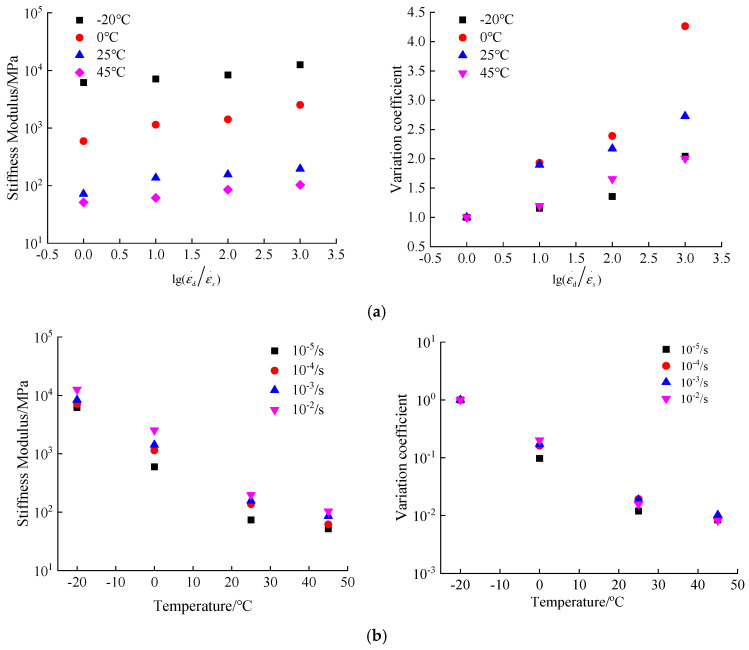
Temperature effect and strain rate effect on the compressive stiffness modulus. (**a**) strain rate effect; (**b**) temperature effect.

Depending on Equation (11), the stiffness modulus of hydraulic asphalt concrete under various strain rates and temperatures is calculated in Figure 9 and Table 3.

The variation coefficient of the stiffness modulus $\alpha_{E}\;$is utilized in the paper to study the strain rate effect on the stiffness modulus of hydraulic asphalt concrete, as presented in Equation (12):

(12) $$\begin{array}{c} \alpha_{S}=\frac{S_{d}}{S_{s}} \end{array}$$

where $S_{d}$ represents the stiffness modulus of concrete at dynamic and static strain rates while $S_{s}$ represents the stiffness modulus of concrete at static strain rate.

Considering the temperature effect on the compressive stiffness modulus of hydraulic asphalt concrete, the stiffness modulus of concrete at temperature −20 °C is taken as the reference value $S_{-20}$ while $S_{0},\;S_{25}$ and $S_{45}$ are taken as the analytical values. The temperature effect on the stiffness modulus of hydraulic asphalt concrete is calculated from the stress-strain curves in Figure 6 and presented in Figure 9b.

According to Equations (11) and (12) and Figure 6, the strain rate effect on the compressive stiffness modulus of hydraulic asphalt concrete is calculated in Figure 9a.

As shown in Figure 9a, the relation between the compressive stiffness modulus and strain rate is presented. From the data in Figure 9a, the stiffness modulus increases significantly with an increasing strain rate. When the temperature is −20 °C, the stiffness modulus of hydraulic asphalt concrete increases from 6149.7 MPa at static strain rate to 12,557.9 MPa at dynamic strain rate 10^−2^/s, with an increase of 2.04 times. Under the temperatures of 0 °C, 25 °C and 45 °C, the compressive stiffness modulus is changed from 591.8, 71.9 and 51.2 MPa with the static strain rate to 2523.6, 196.2 and 102.7 MPa with dynamic strain rate 10^−2^/s, increased by 4.26, 2.73 and 2.00 times, respectively. Those results suggest that when the temperature is 0 °C, the compressive stiffness modulus of hydraulic asphalt concrete is most affected by the strain rate.

Looking at Figure 9b, the stiffness modulus of hydraulic asphalt concrete decreases significantly by an increase in the temperature. Under the static strain rate of 10^−5^/s, the compressive stiffness modulus of hydraulic asphalt concrete decreases from 6149.7 MPa at −20 °C to 51.2 MPa at 45 °C by 99.2%. When the strain rates are 10^−4^/s, 10^−3^/s and 10^−2^/s, the compressive stiffness modulus are reduced from 7095.1, 8345.3 and 12,557.9 MPa at −20 °C to 61.3, 84.8 and 102.7 MPa at 45 °C, and the decline percentage are 99.1%, 99.0% and 99.2%, respectively. In summary, the data mentioned above remarks that the decline percentage of the peak compressive stress of hydraulic asphalt concrete keeps similar with an increasing temperature, influenced by the strain rate effect. Therefore, affected by the temperature effect, the influence of the strain rate effect on the peak compressive stress of hydraulic asphalt concrete is relatively small.

#### 3.4.2. Peak Strain

**Figure 10:**
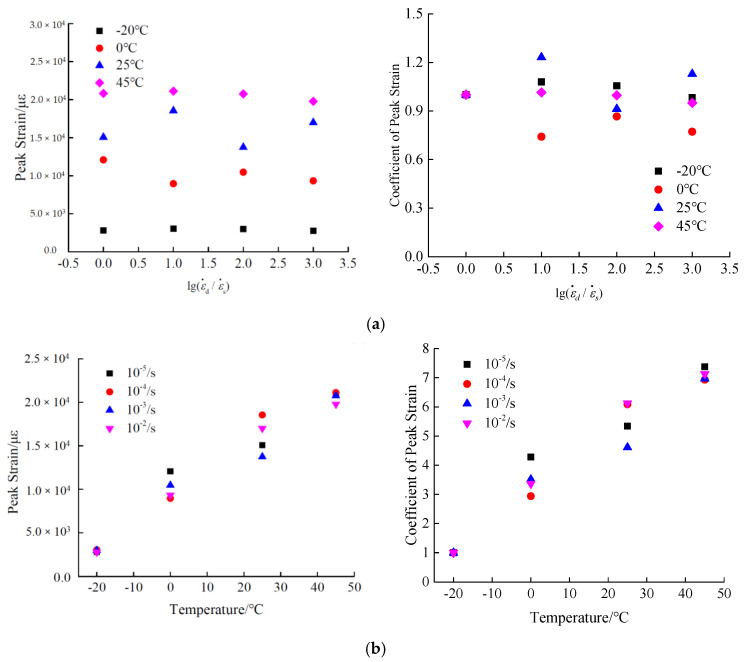
Temperature effect and strain rate effect on the peak strain. (**a**) strain rate effect; (**b**) temperature effect.

The peak strain extracted from the stress-strain curves is used to examine the effect of strain rate and temperature on the peak strain, as presented in Figure 10. For the analysis of the peak strain, the variation coefficient of peak strain is utilized to conduct the research. Similar to the calculation method of variation coefficient of stiffness modulus, the variation coefficient of peak strain is the ratio of the peak strain under dynamic loads to that at static strain rate.

It can be seen in Figure 10 that the peak compressive strain of hydraulic asphalt concrete does not change significantly as the strain rate increases. Compared with the peak strain of quasi-static loading, the range of the peak strain varies from −25.8% to 28.4%. In particular, the dispersion degree of peak strain is large at various strain rates, under temperatures 0 °C and 25 °C. In summary, the trend of the peak strain of hydraulic asphalt concrete is not obviously affected by the strain rate, which is mainly related to the randomness and discreteness of concrete.

According to the data in Figure 10b, there is a significant increase in the peak compressive strain of hydraulic asphalt concrete with the addition of temperatures. Under the static strain rate, the peak compressive strain of hydraulic asphalt concrete is increased from 2822.8 με at temperature −20 °C to 20821.6 με at temperature 45 °C, with an increase of 7.38 times. When the strain rates are 10^−4^/s, 10^−3^/s and 10^−2^/s, the peak compressive strain are increased from 3048.4 με, 2979.8 με and 2775.1 με at −20 °C to 21121 με, 20,758.4 με and 19780.3 με at 45 °C, and the increase ranges are 6.93, 6.97 and 7.13 times, respectively. These results indicate that under various loading strain rates, the peak compressive strain of hydraulic asphalt concrete is significantly increased, affected by temperature effect [34].

## 4. Discussion

The above parts have concluded that the stress-strain curves and peak compressive stress of hydraulic asphalt concrete are closely related to temperature and strain rate. The above expressions have established the mathematical relations between temperature and dynamic increase factor of peak compressive stress, strain rate and dynamic increase factor of peak compressive stress. However, each parameter corresponds to one mathematical relation, and separation equations may cause difficulties in the calculation and application of parameters in the Duncan-Chang model. Therefore, in order to establish the coupling effect model between temperature and strain rate on the peak compressive stress, it is necessary to explore the relevant parameters in the equations mentioned above.

**Figure 11:**
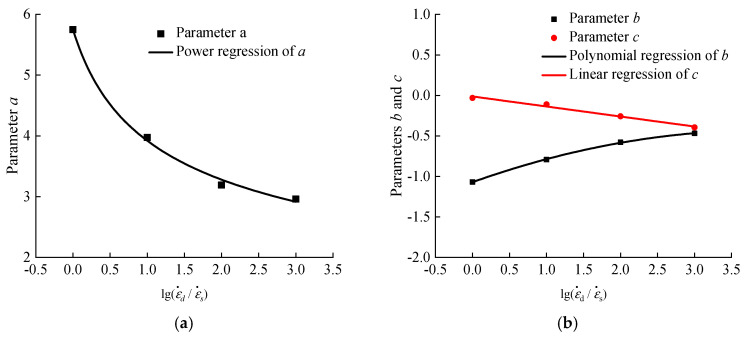
Relation between temperature effect parameters and strain rate. (**a**) parameter *a*; (**b**) parameters *b* and *c*.

Since the temperature of specimens has a power function relation with the variation coefficient of peak compressive stress, as presented in Equations (1)–(4). Based on the parameters extracted from expressions, the relations between the parameters and the logarithmic value of the strain rate ratio after dimensionless treatment are presented in Figure 11, using mathematical regression analysis.

As shown in Figure 11, the relations between the logarithmic value of the strain rate ratio after dimensionless treatment and parameters a, b and c are quadratic, linear and quadratic, respectively. The relevant expressions are obtained as displayed in Equations (13)–(15):

(13) $$\begin{array}{c} a=4.518\times {\left[0.5+\log\left(\frac{\dot{\varepsilon_{d}}}{\dot{\varepsilon_{s}}}\right)\right]}^{-0.3492}\;R^{2}=0.9974 \end{array}$$

(14) $$\begin{array}{c} b=-0.04127\times \log{\left(\frac{\dot{\varepsilon_{d}}}{\dot{\varepsilon_{s}}}\right)}^{2}+0.3253\times \log\left(\frac{\dot{\varepsilon_{d}}}{\dot{\varepsilon_{s}}}\right)-1.07\;R^{2}=0.9996 \end{array}$$

(15) $$\begin{array}{c} c=-0.1238\times \log\left(\frac{\dot{\varepsilon_{d}}}{\dot{\varepsilon_{s}}}\right)-0.01242\;R^{2}=0.9858 \end{array}$$

Substituting Equations (13)–(15) into Equation (1), the model equation of the coupling effect between the temperature and strain rate on the peak compressive stress of hydraulic asphalt concrete could be obtained, as shown in Equation (16):

(16) $$\begin{array}{c} \sigma /\sigma_{-20}=\left\{4.518\times {\left[0.5+\log\left(\frac{\dot{\varepsilon_{d}}}{\dot{\varepsilon_{s}}}\right)\right]}^{-0.3492}\right\}\times {\left(T+25\right)}^{-0.04127\times \log{\left(\frac{\dot{\varepsilon_{d}}}{\dot{\varepsilon_{s}}}\right)}^{2}+0.3253\times \log\left(\frac{\dot{\varepsilon_{d}}}{\dot{\varepsilon_{s}}}\right)-1.07}\\ -0.1238\times \log\left(\frac{\dot{\varepsilon_{d}}}{\dot{\varepsilon_{s}}}\right)-0.01242 \end{array}$$

**Figure 12:**
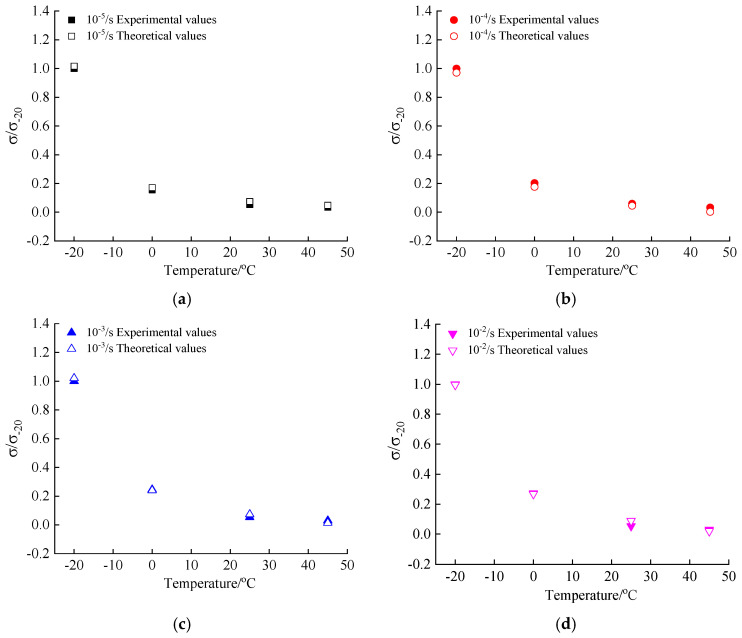
Comparison of theoretical and experimental values of peak compressive stress. (**a**) strain rate 10^−5^/s; (**b**) strain rate 10^−4^/s; (**c**) strain rate 10^−3^/s; (**d**) strain rate 10^−2^/s.

Depending on Equation (16), the theoretical values of the peak compressive stress at different loading conditions are calculated and compared with experimental values in this paper, as presented in Figure 12.

From the data in Figure 12, the comparison results prove that the model equation of the coupling effect proposed in the paper is applicable to reflect the trend of the peak compressive stress of hydraulic asphalt concrete under the influence of temperature and strain rate.

**Figure 13:**
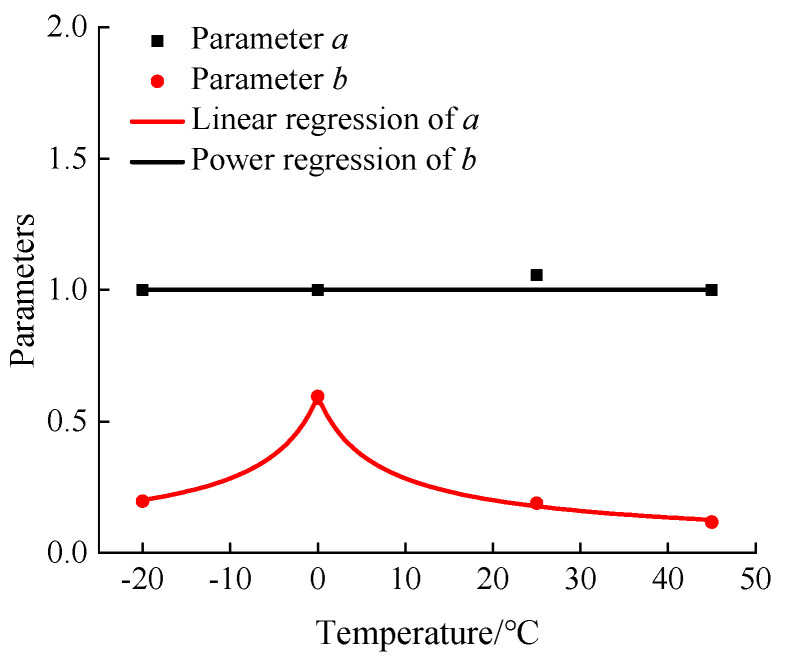
Relation between strain rate effect parameters and temperature.

A combination of quantitative and qualitative approaches is used to analyze the coupling effect between the strain rate and temperature on the dynamic increase factor of the peak compressive stress. The logarithmic value of the strain rate ratio after dimensionless treatment is linearly related to the dynamic increase factor, as shown in Equation (6). The parameters of equations at various temperatures are extracted to generate the relation between parameters and temperatures using the mathematical regression analysis in Figure 13.

According to Figure 13, the parameter a is between 1 and 1.056. Considering that the parameter a is the dynamic increase factor at the static strain rate, the parameter a can be set as 1. The relation between parameter b and temperature is displayed in Figure 13. Therefore, the relevant expressions are obtained as shown below:

(17) $$\begin{array}{c} a=1 \end{array}$$

(18) $$\begin{array}{c} b=1.769\times {\left(\left|T\right|+5\right)}^{-0.6762}\;R^{2}=0.9984 \end{array}$$

Substituting Equations (17) and (18) into Equation (6), the model equation of the coupling effect between temperature and strain rate on the peak stress of hydraulic asphalt concrete in compression is obtained, as shown in Equation (19):

(19) $$\begin{array}{c} \alpha_{DIF}=1+\left[1.769\times {\left(\left|T\right|+5\right)}^{-0.6762}\right]\times \log\left(\frac{\dot{\varepsilon_{d}}}{\dot{\varepsilon_{s}}}\right) \end{array}$$

**Figure 14:**
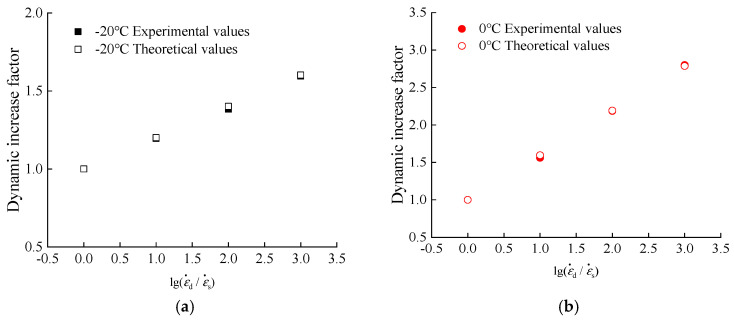

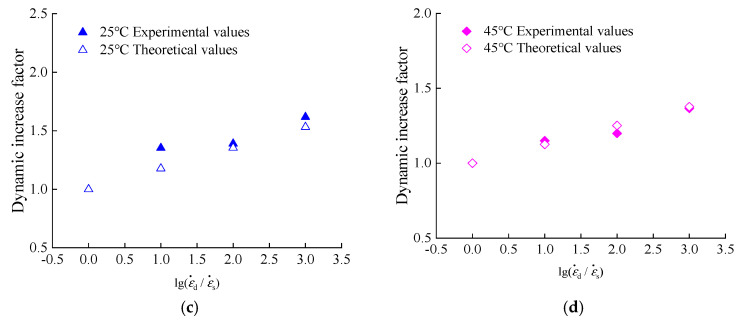
Comparison of theoretical and experimental values of peak compressive stress. (**a**) temperature −20 °C; (**b**) temperature 0 °C; (**c**) temperature 25 °C; (**d**) temperature 45 °C.

The theoretical values of the peak compressive stress of hydraulic asphalt concrete are calculated through Equation (19) and compared with the experimental values in the paper. The comparison results are presented in Figure 14.

From the data in Figure 14, the comparison results prove that the model equation of the coupling effect proposed in the paper is applicable to reflect the trend of the peak compressive stress of hydraulic asphalt concrete under the influence of temperature and strain rate.

In summary, two calculation models proposed in this paper are well applicable to describe coupling effect between the temperature and strain rate on the development of peak stress of hydraulic asphalt concrete under compression. Both models derived above provide the connection and correction for the failure criteria study as well as the basis for establishing the stress-strain constitutive relation of hydraulic asphalt concrete, which is of great engineering and research significance.

## 5. Conclusions

This paper has examined the dynamic behaviors of hydraulic asphalt concrete under four strain rates and four temperatures through uniaxial compression tests. Four strain rates include 10^−5^/s, 10^−4^/s, 10^−3^/s and 10^−2^/s while four temperatures involve −20 °C, 0 °C, 25 °C and 45 °C. These findings are summarized below:

(1) The failure modes of hydraulic asphalt concrete are affected by strain rate and temperature. The integrity of concrete keeps good. The brittle failure of bitumen binder occurs in the specimens at temperatures −20 °C and 0 °C, with small deformation and vertical cracks on the free surfaces. With the rise of the temperature, the bitumen turns from elastic-brittleness to viscoelasticity. The plastic deformation of the specimen gradually increases and the ductile failure occurs. The compressive strain becomes larger and transverse stripes of aggregates on the free surfaces become more obvious.
(2) The stress-strain curves of hydraulic asphalt concrete are closely related to temperature. The slope of stress-strain curves is the largest as well as the peak stress at temperature −20 °C. The stress rise rate is the fastest. Following the addition of the temperature, the stress rise rate decreases with the decline of the peak stress. After the peak stress, hydraulic asphalt concrete exhibits strain softening at −20 °C and 0 °C, and shows strain hardening at 25 °C and 45 °C.
(3) Influenced by strain rate, the trends of stress-strain curves of hydraulic asphalt concrete before the peak point are similar. However, after the peak stress the curves at strain rates 10^−5^/s and 10^−4^/s exhibit strain hardening while curves at strain rates 10^−3^/s and 10^−2^/s show strain softening. By an increase in the strain rate, hydraulic asphalt concrete exhibits more obvious strain softening effect, and the stress descending rate increases.
(4) With successive rise in the temperature, the peak stress of hydraulic asphalt concrete decreases, approaching zero. The relation between the peak stress and temperature fits a power function. At the same time, there is a significant increase in the peak stress with the addition of strain rate. The peak stress is linearly increased with the logarithmic value of the dimensionless-treated strain rate ratio.
(5) Based on the mathematical expressions of temperature, strain rate and peak stress, two model equations of the coupling effect between the temperature and strain rate on the peak compressive stress of hydraulic asphalt concrete are proposed. The model equations are well applicable to describe the trends of peak compressive stress influenced by temperature and strain rate.

## Figures and Tables

**Table 1:** Mix ratio of asphalt concrete (% by weight of total mix).

Concrete Category	Filler Material (<0.075 mm)	Fine Aggregate (0.075–2.36 mm)	Coarse Aggregate (2.36–19 mm)	Bitumen Content (%)
Ordinary asphalt concrete	8	43	49	5
Hydraulic asphalt concrete range	10–15	35–52	33–55	6.5–8.5
Hydraulic asphalt concrete	14.2	37.9	47.9	7.6

**Table 2:** Volumetric properties of hydraulic asphalt concrete and bitumen.

Hydraulic Asphalt Concrete			SBS Modified Bitumen		
Air Voids (%)	VMA ^1^ (%)	VFB ^2^ (%)	Penetration (25 °C)	Ductility (5 °C, 5 mm/min)	Soft Point (°C)
1.6	17.2	90.5	66.5	32.5	72.5

^1^ VMA = volume of air and bitumen in percentage of total volume of specimen; ^2^ VFB = volume of bitumen in percentage of volume of air and bitumen.

**Table 3:** Stiffness modulus of hydraulic asphalt concrete under various loading conditions.

Strain Rate	Temperature	Stiffness Modulus/MPa	Strain Rate	Temperature	Stiffness Modulus/MPa
10^−5^/s	−20 °C	6149.7	10^−3^/s	−20 °C	8345.3
	0 °C	595.8		0 °C	1414.5
	25 °C	73.3		25 °C	156.5
	45 °C	51.2		45 °C	84.8
10^−4^/s	−20 °C	7095.1	10^−2^/s	−20 °C	12,557.9
	0 °C	1142.3		0 °C	2523.6
	25 °C	136.5		25 °C	196.2
	45 °C	61.3		45 °C	102.7
